# Disposal of donor livers in Brazil: how to optimize their utilization rate in transplants?

**DOI:** 10.31744/einstein_journal/2021AO6770

**Published:** 2021-11-22

**Authors:** Júlia Porto de Oliveira Drezza, Amanda Pinter Carvalheiro da Silva Boteon, Igor Lepski Calil, Raoni Salomão Sant Anna, Marcelo de Melo Viveiros, Marcelo Bruno de Rezende, Rafael Antonio Arruda Pecora, Yuri Longatto Boteon

**Affiliations:** 1 Hospital Israelita Albert Einstein Faculdade Israelita de Ciências da Saúde Albert Einstein São Paulo SP Brazil Faculdade Israelita de Ciências da Saúde Albert Einstein, Hospital Israelita Albert Einstein, São Paulo, SP, Brazil.; 2 Hospital Israelita Albert Einstein São Paulo SP Brazil Hospital Israelita Albert Einstein, São Paulo, SP, Brazil.; 3 Hospital Israelita Albert Einstein Instituto Israelita de Ensino e Pesquisa Albert Einstein São Paulo SP Brazil Instituto Israelita de Ensino e Pesquisa Albert Einstein, Hospital Israelita Albert Einstein, São Paulo, SP, Brazil.

**Keywords:** Liver transplantation, Tissue and organ procurement, Organ preservation, Perfusion/methods

## Abstract

**Objective:**

To understand the professionals´ perception of the use of deceased donor liver for transplantation, the reasons to decline them, and propose strategies to increase their use safely.

**Methods:**

This is a cross-sectional, descriptive qualitative-quantitative study. Professionals working with liver transplantation answered a self-administered, structured, and electronic questionnaire comprising 17 questions distributed into four sessions (demographic factors, perception of use of organs, reasons for disposal, and measures to favor their usage).

**Results:**

A total of 42 professionals participated in the study. The rate of use of organs was considered low by 71.43% (n=30) of respondents or very low by 19.05% (n=8). Everyone agreed that it was possible to increase it. Thirty-one (73.81%) participants believed the expansion of the population of extended criteria donors affected this index negatively. Donor-related conditions were the most frequent category of reasons for refusing a liver for transplantation, being the findings during organ retrieval the most frequent reason in clinical practice. Enhanced training of intensive care teams in the treatment of donors was the primary measure selected to favor the use of the organs, followed by investment in new technologies to optimize its preservation/evaluate its function before transplantation.

**Conclusion:**

Implementation of strategies to increase the rate of acceptance of livers is expected. Improvements in donor intensive care and implementation of new preservation technologies should favor the use of the organs.

## INTRODUCTION

Brazil ranks second as countries in absolute number of liver transplants performed. In 2019, the total number of transplants reached 23,957, of which 2,245 were liver transplants.^(1)^ Between 2009 and 2019, there was an increase in the number of liver transplants (from 1,603 to 2,245), as well as of deceased donors (2,406 in 2012, to 3,768) and teams specialized in performing the procedure (59 in 2009, to 74 in 2019). Despite these successful figures, there is a persistent disparity between the number of transplants performed and the number needed - which, in 2019, was 2,967 transplants.^(1)^

This disparity is exacerbated by the current utilization rate of deceased donor livers. In 2019, out of 3,768 effective deceased donors, only 2,245 livers were transplanted.^(1)^ This number is in line with international literature, where disposal rates of donor livers for transplantation are over 30%.^(2)^ The obesity epidemic, aging population, and increasing prevalence of chronic diseases are also reflected in organ donors.^(3,4)^ Non-transplanted livers are often from donors with advanced age, higher body mass index (BMI), carriers of viral hepatitis (B and C viruses), and a higher number of comorbidities.^(5)^ The organs from these non-ideal donors or extended criteria donors are at increased risk for postoperative complications and even primary graft non-function.^(4,5)^ Consequently, expansion of this extended criteria donor population compromises utilization rates for these organs.^(4)^

However, organs from donors with borderline characteristics, which would not previously have been considered for donation, began to be transplanted to meet the demand.^(6)^ Initiatives to favor the safe use of these high-risk organs include optimizing the intensive care of donors. It is noteworthy, though, that while this initiative mitigates a worsening of organ damage, reversal of unfavorable demographic characteristics of this population (*e.g*., obesity and senility) is not possible. Thus, the implementation of strategies to evaluate the metabolic capacity of these livers before transplantation and potentially their reconditioning, has gained increasing attention from the transplant community.^(7,8)^

Although it is possible to gather general data about organ transplantation and donation in Brazil, an investigation about the professionals´ perception of the utilization rate of these livers, the most frequent reasons for their refusal, and the proposition of measures for their safe optimization is still pending.

## OBJECTIVE

To understand the professionals´ perception of the use of deceased donor liver for transplantation, the reasons to decline them, and propose strategies to increase their use safely.

## METHODS

### Study design, selection, and recruitment of participants

This is a cross-sectional, descriptive qualitative-quantitative study, using a self-administered, structured, electronic questionnaire for data collection. The questionnaire was applied between February and March 2021. The project was approved by the Research Ethics Committee of *Hospital Israelita Albert Einstein* (HIAE), with opinion 4.528.519, CAAE: 40071220.0000.0071. All participants confirmed their acceptance to participate in the study by means of an Informed Consent Form (ICF) available online. Each respondent received a signed, dated, and initialed copy from the researcher in charge at their e-mail address.

Professionals who worked with liver transplantation in Brazil were approached virtually by e-mail and messages. The survey questionnaire was made available over the Internet.

The inclusion criterion for participants in the study was the health professional working with liver transplantation in centers that used deceased organ donors. The exclusion criteria were professionals working exclusively with living donors and those who did not participate in the process of choosing organs for transplantation.

### Structured questionnaire

The questionnaire was created in SurveyMonkey^®^ 14 software, and structured with 17 questions distributed into four sections (Appendix 1) Section I - Demographic factors; Section II - Perception of use of livers from deceased donors; Section III - Reasons for discarding livers from donors for transplantation; and Section IV Measures to favor the use of livers from donors. The closed and multiple-choice questions addressed the consensual reasons established for the analysis of the outcomes researched and composed the quantitative portion of the study. Questions with open fields, in which the participant could report other data, were included in the questionnaire for its qualitative part.

We chose to collect data via an electronic form applied over the internet to favor the national scope of the study, facilitate contact with the participants, and offer them the possibility of answering the questionnaire at the appropriate time.

### Statistical analysis

Categorical variables were described and analyzed; absolute numbers and frequency (percentage) were used. For qualitative data, the results were presented in the form of reports that focused on the interviewees’ point of view. Statistical analysis tests were performed using (SPSS) software, version 22 (IBM Corp, Armonk, New York).

## RESULTS

### Demographic data of respondents

Forty-two participants responded to the research. Most participants (26; 61.90%) worked in transplant centers in the Southeast Region of the country and had more than five years of experience in liver transplantation (38; 90.48%). Moreover, the transplant centers to which the respondents belonged performed more than 60 transplants per year (24; 57.14%). The detailed demographic distribution of the respondents is shown in table 1.

**Table 1:** Demographic characteristics of the 42 study participants

Characteristics		n (%)
Region of the transplant center		
	North	2 (4.76)
	Northeast	7 (16.67)
	South	6 (14.29)
	Southeast	26 (61.90)
	Midwest	1 (2.38)
Working with liver transplantation, years		
	<5	4 (9.52)
	5-10	13 (30.95)
	10 -15	10 (23.81)
	>15	15 (35.72)
Transplants/year at the service		
	<30	11 (26.19)
	30-60	7 (16.67)
	>60	24 (57.14)

### Perception of use of livers from deceased donors

**Figure 1:**
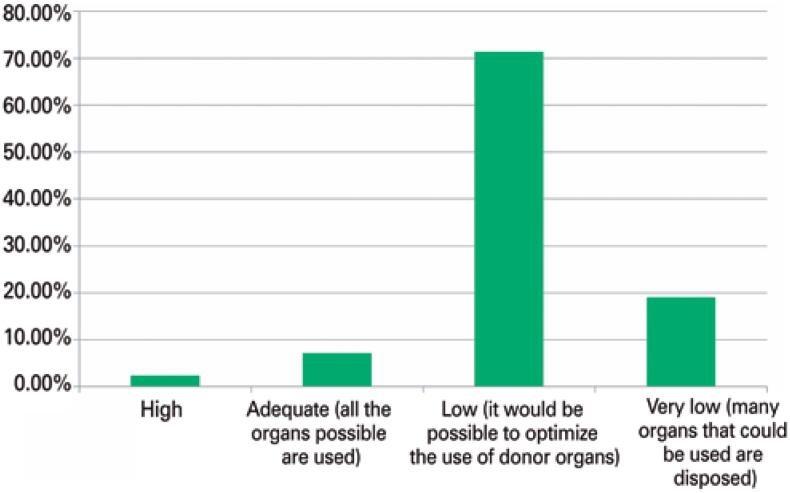
Respondents’ opinions regarding their perception of the utilization rate of deceased donor livers (ratio between the number of organs offered and the number of organs transplanted) in Brazil

When asked about their perception of the utilization rate of deceased donor livers for transplantation in Brazil (ratio between the number of organs offered and those transplanted), the absolute majority of participants (38; 90%) believed it was low or very low (Figure 1). All participants answered it is possible to increase the utilization rate of livers from deceased donors in the country.

**Figure 2:**
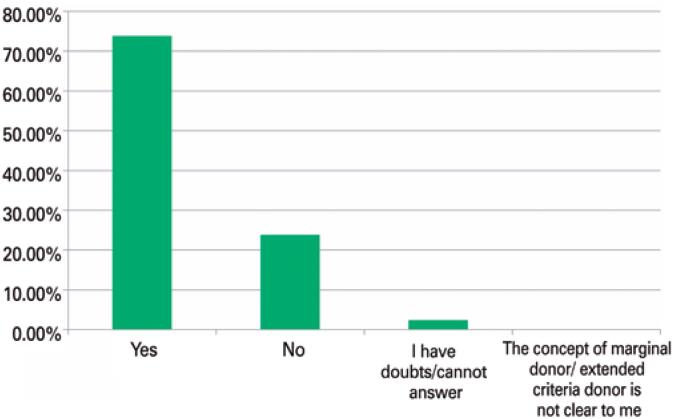
Participants’ perceptions of the negative influence of the expansion of the extended criteria donor population on the utilization rate of livers from deceased donors

The majority of participants (36; 85.71%) believed that, over the past few years, the prevalence of marginality criteria among organ donors offered for transplantation had increased, with 9.52% (n=4) saying no, and 4.76% (n=2) being in doubt. In parallel, 72% (n=30) of participants believed the expansion of the extended criteria donor population negatively affected the organ utilization rate. The data is presented in figure 2.

### Reasons for discarding deceased donor livers for transplantation

**Figure 3:**
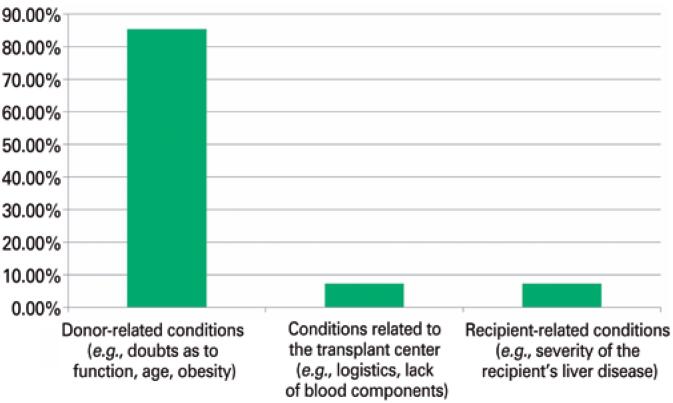
Participants’ answers regarding the main categories of reasons for refusing a deceased donor liver for transplantation

The participants were also asked about the main category of reasons for refusing an organ for transplantation. Most respondents (36; 85.37%) considered donor-related conditions (doubts regarding function, age, obesity, etc.) as the main category. Figure 3 shows all the questioned categories, as well as the distribution of the participants’ answers.

Next, they were asked about the frequency in clinical practice of the reasons for not accepting a deceased donor liver for transplantation. Nineteen participants (46%) selected findings during harvesting (steatosis, anatomical abnormalities, and injuries during harvesting, unsatisfactory organ perfusion, and organ size - donor/recipient disproportion) as the first reason. Problems related to cold ischemia time and difficulty finding a good match between donor and recipient were the next most frequent reasons (Table 2).

**Table 2:** Main reasons in clinical practice selected by participants for refusing a deceased donor liver, in order of frequency

Reason	n (%)
Findings during harvesting (steatosis, anatomical abnormalities, injuries during harvesting, unsatisfactory organ perfusion, and organ size - donor/recipient disproportion)	19 (46.0)
Prolonged estimated cold ischemia time (due to the long distance from the organ procurement hospital to the transplant center)	14 (34.0)
Difficulty in making a good match between donor and recipient (balance between the severity of liver disease of the recipient and characteristics of the donor/organ)	15 (37.0)

Other reasons not included in the questionnaire options that participants highlighted were difficulty in interpreting the organ information due to inexperience of the professional responsible for harvesting, donor's hemodynamic instability, difficulty in obtaining air logistics for transporting the donor's liver, and infectious and contagious conditions of the donor (including positive serology for Chagas disease).

### Measures to favor the use of livers from deceased donors

In order of relevance, the participants were asked about the list of possible measures to optimize the use of livers from deceased donors for transplantation. Of the measures suggested to the participants, improved training of intensive care unit (ICU) staff in organ donor care was listed as the most relevant by 53.66% (n=22) of respondents. The development/investment in new technologies was the second measure. The results are displayed in table 3.

**Table 3:** List, by order of relevance, as per participants, of the main measures to optimize the use of deceased donor livers

Measure	n (%)
First measure: better training of intensive care unit staff in organ donor care	22 (53.66)
Second measure: development/investment in new technologies to improve preservation and perform function evaluation of donor organs before transplantation (machine perfusion of the liver)	22 (53.66)

Regarding the potential new technologies to favor greater use of donor's livers, the participants were questioned regarding their perception of a likely positive impact of the machine perfusion of the liver. Most of them (31; 75.61%) believed in this device, with 12.20% (n=5) responding negatively and 12.20% (n=5) selecting that they did not have a formed opinion.

Twenty-seven (65.85%) participants selected other strategies not listed in the options that could optimize the utilization rate of livers from deceased donors. The options suggested were performing the harvesting surgeries by experienced professionals (especially in extended criteria donors); creating an alternative list dedicated to oncology patients for organs discarded by all teams; greater availability of resources to perform liver biopsy at the donor's hospital; decreasing consultation time for organ evaluation by the teams; establishing a national policy for split organ utilization; establishing audits of the harvesting surgeries and causes of organ disposal with the definition of criteria for registration and maintenance of transplant centers; facilitating air logistics for the transportation of the donor's liver, and promotion of a better understanding of the balance of risks between donor and recipient, which should favor the use of organs from higher-risk donors.

## DISCUSSION

Developing and implementing strategies to improve the utilization rate of deceased donor organs are fundamental to allow more people to have their lives saved and transformed through liver transplantation. In this questionnaire study involving professionals working with liver transplantation, it was found that most professionals considered the utilization rate of livers from deceased donors in Brazil low or very low and unanimously agreed it was possible to increase it. Donor-related conditions was the most frequent category of reasons for refusing a liver for transplantation, with findings during harvesting being the most frequent reason in clinical practice. Better training of ICU staff in organ donor care and investment in new technologies to optimize organ preservation/evaluate organ quality before transplantation were the most commonly considered measures to increase the utilization rate of deceased donor organs safely.

A recent study analyzed data from the Scientific Registry of Transplant Recipients (SRTR). In it, Carpenter *et al.,* identified that non-transplanted livers were more frequently from older donors, with higher BMI, viral hepatitis (B and C viruses), and a higher number of comorbidities, revealing doubts regarding the quality of the organ.^(5)^ In this analysis of 6,454 livers from brain dead donors discarded for transplantation, the main reason for disposal was biopsy findings, followed by “others,” *i.e.*, doubts regarding organ function, anatomical alterations of the organs, clinical worsening of the recipient's disease, prolonged cold ischemia time, and lack of a compatible recipient.^(5)^

Similar findings were reported in another study using the United Network for Organ Sharing (UNOS) database of 9,021 discarded livers.^(9)^ The most common cause for discarding organs from deceased donors for transplantation was biopsy findings (4,069 organs). This reason was followed by “other specific reasons”, reported for 1,456 organs. The authors pointed out that, considering these readings are commonly performed by non-specialized pathologists, standardization of reporting may allow an increase in the utilization rate of these organs. Other reasons for not accepting an organ were inability to find a suitable recipient, social history, positive serology for cytomegalovirus or viral hepatites, donor medical history, absence of adequate clinical condition of the donor, vascular lesions, trauma, and anatomical abnormalities.^(9)^

Recently, a single-center retrospective study in Brazil reviewed data from 67 deceased donor livers discarded for transplantation between 2015 and 2018 after harvesting.^(10)^ Problems related to the donor organ were the cause for not using half of the organs (macroscopic pathological changes, visible organ damage, and inappropriate size), and 36% were associated with clinical and laboratory characteristics of the donor (positive hepatitis B virus serology, infection in the donor, past medical history of the donor, prolonged use of vasopressors, and hemodynamic instability). Six organs (9%) were discarded for logistical reasons and cold ischemia time.^(10)^

The 2018 and 2019 Brazilian Transplant Registry (RBT) records described cerebrovascular diseases as the leading cause of death (55%) in the deceased donor population - no longer traumatic causes.^(1,11)^ Also noteworthy is the fact that 47% of these donors are aged over 50 years, and in 2019, there was a 62.5% increase in the rate of donors over 65 years.^(1,11)^ As was discussed, donors who do not meet the standards of an ideal donor are known as extended criteria donors. The prevalence of these high-risk donors has increased over the years because of demographic change in this population, and they are increasingly being offered for transplantation.^(12)^ Donors are older, often obese, have chronic diseases, and die from cerebrovascular diseases, evidence also found in international studies.^(3,4)^ These organs are at higher risk of postoperative complications and consequently more often refused for transplantation.^(4,12)^

The complexity of the potential organ donor is widely recognized as a challenge in intensive care, considering the multiplicity of their physiological priorities to remain eligible for donation, in which hemodynamic stability is the main success factor.^(13)^ In view of this, a task force of societies and associations linked to organ transplantation in Brazil sought to develop and disseminate protocols for managing the potential donor in the ICU.^(14)^ Although crucial, optimization of care of the potential donor cannot change the patient's biological characteristics, such as age, weight, and comorbidities, nor reverse previous aggressions to the organs.

The present study portrays the need to expand the use of livers from deceased donors for transplantation in Brazil, which is compromised by the prevalence of donors with extended criteria. Moreover, the main reason for their refusal is findings during donor harvesting, which generate doubts as to the quality of the organ. Given this scenario, the participants considered the development/investment in new technologies to improve organ preservation and evaluate organ function before transplantation (machine perfusion of the liver). This measure could increase this utilization rate. Dynamic organ preservation via the machine perfusion of the liver offers greater organ preservation from extended criteria donors and allows reconditioning and evaluation of organ function before transplantation.^(7,15,16)^ The continuous flow of an oxygenated solution through the vasculature prevents ischemic damage to the organ, removes toxic metabolites, and allows the evaluation of its metabolic activity.^(17)^ Currently, this technology is being implemented for clinical use in countries such as the United Kingdom, Switzerland, and Spain.^(15,16,18)^ In Brazil, careful donor selection and adequate technical-scientific knowledge can guarantee the effective and satisfactory implementation of this technology.^(19)^

Among the strategies to increase the use of deceased donor organs proposed by the participants, improvements in donor organ procurement are highlighted, such as the allocation of experienced professionals for this procedure (especially in extended criteria donors), and the facilitation of liver biopsy performance in the donor's hospital and air logistics for organ transportation. Other suggested initiatives focused on the field of transplant policies, the creation of an alternative list dedicated to oncologic patients for organs discarded by all teams, reduction of consultation time for donor evaluation, creation of a national policy for the use of split organs, and audits of the harvesting operations and the causes of organ disposal, with the definition of criteria for the registration and maintenance of transplant centers. Finally, dissemination of the concept of risk balance between donor and recipient should favor the use of organs from higher-risk donors.

All suggested measures are indeed of great value and should favor optimizing the utilization rate of livers from deceased donors. Although many of these initiatives depend on regulatory agencies and external funding, simple attitudes of transplant teams, such as the designation of experienced professionals for harvesting and the rapid evaluation of donor offers, can favor the use of these organs. The creation of support groups for access to new technology and transplant education are other examples of beneficial measures. Ensuring that transplant teams have the support and resources they need is fundamental to increasing the use of deceased donor organs, especially those at higher risk.

Among the limitations of this study is the number of participants, although its distribution was representative of the location of transplant centers throughout the Brazilian geographic regions. To mitigate the limitation arising from the application of a questionnaire with closed multiple-choice answers, the study presented a qualitative-quantitative design, allowing the participants to provide answers in open fields.

## CONCLUSION

Among health professionals directly involved in liver transplantation in Brazil, there is the perception of a low utilization rate of organs from deceased donors. The compromised utilization rate may be a consequence of donor-related conditions. Safely increasing organ utilization rate is fundamental to saving more lives. The commitment and joint work of the teams involved in the donation and transplantation process with this common purpose are essential to achieving this goal. The improvement of intensive care of organ donors and the implementation of new technologies that allow better preservation and quality assessment of livers from extended criteria donors before transplantation were indicated in this study as the most relevant measures.

## Annex 1 Questionnaire

**Table:**

Section I – Demographic factors	
1. In which region of Brazil is your transplant center located?	
	a) North
	b) Northeast
	c) South
	d) Southwest
	e) Midwest
2. How many years have you been working specifically with liver transplantation?	
	a) <5 years
	b) 5-10 years
	c) 10-15 years
	d) >15 years
3. How many liver transplants are performed at your center annually?	
	a) <30
	b) 30-60
	c) >60
Section II – Perception of the use of livers from deceased donors	
4. How do you consider the utilization rate of livers from deceased donors (ratio between the number of organs offered and the number of organs transplanted) today in Brazil?	
	a) High.
	b) Appropriate (all organs possible are utilized).
	c) Low (it would be possible to optimize the utilization of donor organs).
	d) Very low (many organs that could be utilized are discarded).
5. Do you think it is possible to increase the utilization rate of livers from deceased donors currently practiced in Brazil?	
	a) Yes
	b) No
6. Do you believe that over the past few years there has been a higher prevalence of marginality criteria among organ donors offered for transplantation?	
	a) Yes
	b) No
7. Do you believe the expansion of the extended criteria donor population has directly affected the utilization rate of donor livers?	
	a) Yes
	b) No
Section III – Reasons for discarding donor livers for transplantation	
8. Into which of the following categories do your reasons for not accepting a donor organ for transplantation most commonly fall?	
	a) Donor related conditions (*e.g.*, doubts regarding function, age, and obesity).
	b) Conditions related to the transplant center (for example, logistics, and lack of blood components).
	c) Recipient-related conditions (*e.g.*, severity of the recipient's liver disease).
9. Among the reasons below for not accepting an organ (liver) from a deceased donor, which do you consider the major factor, in your clinical practice?	
	a) Prolonged estimated cold ischemia time (due to the long distance from the organ procurement hospital to the transplant center).
	b) Difficulty in making a good match between donor and recipient (balance between the recipients’ liver disease severity and donor/organ characteristics).
	c) Donor past medical history data (including laboratory tests) - doubts regarding organ function.
	d) Findings during harvesting (steatosis, anatomical abnormalities, injuries during the surgery, unsatisfactory organ perfusion, and organ size - donor/recipient disproportion).
	e) Donor organ biopsy findings.
10. Among the reasons below for not accepting an organ (liver) from a deceased donor, which do you consider the second major factor in your clinical practice?	
	a) Prolonged estimated cold ischemia time (due to the long distance from the organ procurement hospital to the transplant center).
	b) Difficulty in making a good match between donor and recipient (balance between the liver disease severity of recipients and donor/organ characteristics).
	c) Donor past medical history data (including laboratory tests) - doubts regarding organ function.
	d) Findings during the extraction (steatosis, anatomical abnormalities, injuries during extraction, unsatisfactory organ perfusion, and organ size - donor/recipient disproportion).
	e) Donor organ biopsy findings.
11. Among the reasons below for not accepting an organ (liver) from a deceased donor, which do you consider the third major motive in your clinical practice?	
	a) Prolonged estimated cold ischemia time (due to the long distance from the organ procurement hospital to the transplant center).
	b) Difficulty in making a good match between donor and recipient (balance between the recipients’ liver disease severity and donor/organ characteristics).
	c) Donor past medical history data (including laboratory tests) - doubts regarding organ function.
	d) Findings during the harvesting (steatosis, anatomical abnormalities, injuries during the surgery, unsatisfactory organ perfusion, and organ size - donor/recipient disproportion).
	e) Donor organ biopsy findings.
12. In your opinion, are there any other reasons for not accepting an organ (liver) from a deceased donor that are not listed in the options above? If yes, please specify.	
Answer:_________________________________________________	
Section IV – Measures to favor the use of livers from deceased donors	
13. Among the measures below, which do you think would be the primary action to optimize the use of livers from deceased donors? (Select two alternatives)	
	a) Offers only from donors within more restrictive criteria (for example, maximum age limit, serum sodium level at or near normal limits, and body mass index limits).
	b) Development of an organ allocation system that already considers donor and recipient characteristics prior to donation.
	c) Development/investment in new technologies to improve preservation and perform function evaluation of donor organs before transplantation (machine perfusion of the liver).
	d) Better training of intensive care unit teams in organ donor care.
	e) Allocation of organs only regionally (allocation zones), imposing distance limits for organ supply.
14. Among the measures below, which do you think would be the second major action to optimize the use of livers from deceased donors? (Select two alternatives)	
	a) Offers only from donors within more restrictive criteria (*e.g.*, maximum age limit, serum sodium level at or near normal limits, and body mass index limits).
	b) Development of an organ allocation system that already considers donor and recipient characteristics prior to donation.
	c) Development/investment in new technologies to improve preservation and perform function evaluation of donor organs before transplantation (liver perfusion machine).
	d) Better training of intensive care unit teams in organ donor care.
	e) Procurement of organs only regionally (procurement zones), imposing distance limits for organ supply.
15. Do you believe that the implementation of the *ex situ* machine perfusion of the liver will positively affect liver transplantation, favoring a greater use of donor livers?	
	a) Yes
	b) No
	c) I do not have an opinion.
16. Are there other strategies that you think could further improve the donor utilization rate?	
	a) Yes
	b) No
17. If you answered yes to the previous question, please specify which strategy you think would also contribute:	
Answer:____________________________________________________	

## References

[1] Registro Brasileiro de Transplantes (RBT). Veículo Oficial da Associação Brasileira de Transplante de Órgãos (ABTO). Dimensionamento dos transplantes no Brasil e em cada estado (2012-2019). São Paulo: RBT; 2020 [citado 2021 Jun 24]. Disponível em: http://www.abto.org.br/abtov03/Upload/file/RBT/2019/RBT-2019-leitura.pdf

[2] Kim WR, Smith JM, Skeans MA, Schladt DP, Schnitzler MA, Edwards EB, et al. OPTN/SRTR 2012 Annual Data Report: liver. Am J Transplant. 2014;14 Suppl 1:69-96. 10.1111/ajt.12581 24373168

[3] Spitzer AL, Lao OB, Dick AA, Bakthavatsalam R, Halldorson JB, Yeh MM, et al. The biopsied donor liver: incorporating macrosteatosis into high-risk donor assessment. Liver Transpl. 2010;16(7):874-84. 10.1002/lt.22085 20583086

[4] Feng S, Goodrich NP, Bragg-Gresham JL, Dykstra DM, Punch JD, DebRoy MA, et al. Characteristics associated with liver graft failure: the concept of a donor risk index. Am J Transplant. 2006;6(4):783-90. Erratum in: Am J Transplant. 2018;18(12):3085. 10.1111/j.1600-6143.2006.01242.x 16539636

[5] Carpenter DJ, Chiles MC, Verna EC, Halazun KJ, Emond JC, Ratner LE, et al. Deceased brain dead donor liver transplantation and utilization in the United States: nighttime and weekend effects. Transplantation. 2019; 103(7):1392-404. 10.1097/TP.0000000000002533 PMC7096151 30444802

[6] Schlegel A, Kalisvaart M, Scalera I, Laing RW, Mergental H, Mirza DF, et al. The UK DCD Risk Score: a new proposal to define futility in donation-after-circulatory-death liver transplantation. J Hepatol. 2018;68(3):456-64. 10.1016/j.jhep.2017.10.034 29155020

[7] Mergental H, Laing RW, Kirkham AJ, Perera MT, Boteon YL, Attard J, et al. Transplantation of discarded livers following viability testing with normothermic machine perfusion. Nat Commun. 2020;11(1):2939. 10.1038/s41467-020-16251-3 PMC7298000 32546694

[8] van Rijn R, Schurink IJ, de Vries Y, van den Berg AP, Cortes Cerisuelo M, Darwish Murad S, et al. Hypothermic machine perfusion in liver transplantation - a randomized trial. N Engl J Med. 2021:384(15):1391-401. 10.1056/NEJMoa2031532 33626248

[9] Desai C, Khan K, Girlanda R, Hawksworth J, Serrano P, Island E, et al. UNOS data analysis of discarded liver-grafts after procurement. Abstract# 1465. Transplantation. 2014;98(Suppl 1):11.

[10] Bicudo de Oliveira L, Riccetto E, Boin IF. Prevalence and profile of discarded liver donors in a tertiary health service in Brazil From 2015 to 2018. Transplant Proc. 2020;52(5):1251-5. 10.1016/j.transproceed.2020.01.078 32224015

[11] Registro Brasileiro de Transplantes (RBT). Veículo Oficial da Associação Brasileira de Transplante de Órgãos (ABTO). Dimensionamento dos transplantes no Brasil e em cada estado (2011-2018). São Paulo: RBT; 2018 [citado 2021 Jun 24]. Disponível em: www.abto.org.br/abtov03/Upload/file/RBT/2018/Lv_RBT-2018.pdf

[12] Vodkin I, Kuo A. Extended criteria donors in liver transplantation. Clin Liver Dis. 2017;21(2):289-301. Review. 10.1016/j.cld.2016.12.004 28364814

[13] Dare AJ, Bartlett AS, Fraser JF. Critical care of the potential organ donor. Curr Neurol Neurosci Rep. 2012;12(4):456-65. Review. 10.1007/s11910-012-0272-9 22618126

[14] Westphal GA, Robinson CC, Cavalcanti AB, Gonçalves AR, Guterres CM, Teixeira C, et al. Brazilian guidelines for the management of brain-dead potential organ donors. The task force of the AMIB, ABTO, BRICNet, and the General Coordination of the National Transplant System. Ann Intensive Care. 2020;10(1):169. 10.1186/s13613-020-00787-0 PMC7736434 33315161

[15] Nasralla D, Coussios CC, Mergental H, Akhtar MZ, Butler AJ, Ceresa CD, Chiocchia V, Dutton SJ, García-Valdecasas JC, Heaton N, Imber C, Jassem W, Jochmans I, Karani J, Knight SR, Kocabayoglu P, Malagò M, Mirza D, Morris PJ, Pallan A, Paul A, Pavel M, Perera MT, Pirenne J, Ravikumar R, Russell L, Upponi S, Watson CJ, Weissenbacher A, Ploeg RJ, Friend PJ; Consortium for Organ Preservation in Europe. A randomized trial of normothermic preservation in liver transplantation. Nature. 2018;557(7703):50-6. 10.1038/s41586-018-0047-9 29670285

[16] Schlegel A, Muller X, Kalisvaart M, Muellhaupt B, Perera M, Isaac JR, et al. Outcomes of DCD liver transplantation using organs treated by hypothermic oxygenated perfusion before implantation. J Hepatol. 2019;70(1):50-7. 10.1016/j.jhep.2018.10.005 30342115

[17] Boteon YL, Afford SC. Machine perfusion of the liver: Which is the best technique to mitigate ischaemia-reperfusion injury? World J Transplant. 2019;9(1):14-20. 10.5500/wjt.v9.i1.14 PMC6347667 30697517

[18] Pérez Redondo M, Alcántara Carmona S, Fernández Simón I, Villanueva Fernández H, Ortega López A, Pardo Rey C, et al. Implementation of a mobile team to provide normothermic regional perfusion in controlled donation after circulatory death: pilot study and first results. Clin Transplant. 2020;34(8):e13899. 10.1111/ctr.13899 32383200

[19] Boteon YL, Boteon AP. Prospects for the ex situ liver machine perfusion in Brazil. Rev Col Bras Cir. 2020;47:e20202610. 10.1590/0100-6991e-20202610 33053064

