# Supplementary material for: Disposal of donor livers in Brazil: how to optimize their utilization rate in transplants?
Source: Einstein (Sao Paulo). 2021 Nov 22;19:eAO6770. doi: 10.31744/einstein_journal/2021AO6770 (PMC8670423; doi:10.31744/einstein_journal/2021AO6770)
Supplement: Translation

# Translation: Descarte de fígados de doadores no Brasil: como otimizar sua taxa de utilização em transplantes?

**Authors:** Júlia Porto de Oliveira Drezza, Amanda Pinter Carvalheiro da Silva Boteon, Igor Lepski Calil, Raoni Salomão Sant Anna, Marcelo de Melo Viveiros, Marcelo Bruno de Rezende, Rafael Antonio Arruda Pecora, Yuri Longatto Boteon

**Affiliations:** 1 São Paulo SP Brasil Faculdade Israelita de Ciências da Saúde Albert Einstein, Hospital Israelita Albert Einstein, São Paulo, SP, Brasil.; 2 São Paulo SP Brasil Hospital Israelita Albert Einstein, São Paulo, SP, Brasil.; 3 São Paulo SP Brasil Instituto Israelita de Ensino e Pesquisa Albert Einstein, Hospital Israelita Albert Einstein, São Paulo, SP, Brasil.

**Keywords:** Transplante de fígado, Obtenção de tecidos e órgãos, Preservação de órgãos, Perfusão/métodos

## Abstract

**Objetivo:**

Compreender a percepção dos profissionais acerca do uso de fígado de doadores falecidos para transplante e os motivos para sua recusa, além de propor estratégias para ampliar sua utilização com segurança.

**Métodos:**

Estudo do tipo transversal, descritivo qualiquantitativo.Profissionais que trabalhavam com transplante hepático responderam a um questionário autoaplicável, estruturado e eletrônico, composto de 17 questões distribuídas em quatro seções (fatores demográficos, percepção sobre a utilização dos órgãos, razões para o descarte e medidas para favorecer sua utilização).

**Resultados:**

Participaram do estudo 42 profissionais. A taxa de utilização dos órgãos foi considerada baixa por 71,43% (n=30) dos respondentes ou muito baixa por 19,05% (n=8). Todos concordaram que era possível aumentá-la. Trinta e um (73,81%) participantes acreditavam que a expansão da população de doadores de critérios estendidos impacta negativamente nesse índice. Condições relacionadas ao doador foi a categoria mais frequente de razões para a recusa de um fígado para transplante, sendo os achados durante a cirurgia de extração o motivo mais frequente na prática clínica. O melhor treinamento das equipes da terapia intensiva nos cuidados com os doadores foi a principal medida selecionada para favorecer a utilização dos órgãos, seguido pelo investimento em novas tecnologias para otimizar sua preservação/avaliar sua função antes do transplante.

**Conclusão:**

A efetivação de estratégias para aumentar a taxa de aceite de fígados de doadores é aguardada. Melhorias no cuidado intensivo do doador e a implementação de novas tecnologias de preservação devem favorecer a utilização desses órgãos.

## INTRODUÇÃO

O Brasil é o segundo em número absoluto de transplantes hepáticos realizados. No ano de 2019, o número total de transplantes atingiu 23.957, dos quais 2.245 foram de fígado.^(1)^ Entre 2009 e 2019, houve aumento no número de transplantes hepáticos (de 1.603 para 2.245), assim como de doadores falecidos (2.406, em 2012, para 3.768) e de equipes especializadas na realização do procedimento (59, em 2009, para 74, em 2019). Apesar desses números de sucesso, há uma persistente disparidade entre o número de transplantes realizados e o necessário – que, no ano de 2019, era de 2.967 transplantes.^(1)^

Tal disparidade é agravada pela atual taxa de utilização de fígados de doadores falecidos. Em 2019, dos 3.768 doadores falecidos efetivos, apenas 2.245 fígados foram transplantados.^(1)^ Esse número está em acordo com a literatura internacional, segundo a qual as taxas de descarte de fígados de doadores para transplante atingem mais de 30%.^(2)^ A epidemia de obesidade, o envelhecimento da população e o aumento da prevalência de doenças crônicas também se refletem nos doadores de órgãos.^(3,4)^ Fígados não transplantados são frequentemente de doadores com idade avançada, com maiores índices de massa corporal (IMC), portadores de hepatites virais (vírus B e C) e com maior número de comorbidades.^(5)^ Os órgãos desses doadores não ideais ou de doadores de critérios estendidos apresentam risco aumentado de complicações pós-operatórias e mesmo de não função primária do enxerto.^(4,5)^ Consequentemente, a expansão dessa população de doadores de critérios estendidos compromete as taxas de utilização desses órgãos.^(4)^

Porém, para atender a demanda, órgãos de doadores com características limítrofes, que não seriam previamente considerados para doação, passaram a ser transplantados.^(6)^ Iniciativas para favorecer a utilização desses órgãos de alto risco com segurança passam pela otimização dos cuidados de terapia intensiva com os doadores. Cabe ressaltar, porém, que, apesar dessa iniciativa mitigar um agravamento da lesão aos órgãos, a reversão de características demográficas desfavoráveis dessa população (por exemplo: obesidade e senilidade) não é possível. Assim, a implementação de estratégias de avaliação da capacidade metabólica desses fígados antes do transplante, bem como potencialmente de seu recondicionamento, tem ganhado crescente atenção da comunidade transplantadora.^(7,8)^

Embora seja possível a obtenção de dados gerais sobre o transplante e a doação de órgãos no Brasil, uma investigação sobre a percepção dos profissionais acerca da taxa de utilização desses fígados, os motivos mais frequentes para sua recusa e a proposição de medidas para sua otimização com segurança continua pendente.

## OBJETIVO

Compreender a percepção dos profissionais acerca do uso de fígado de doadores falecidos para transplante e os motivos para sua recusa, além de propor estratégias para ampliar sua utilização com segurança.

## MÉTODOS

### Desenho do estudo e seleção e abordagem dos participantes

Estudo do tipo transversal, de metodologia qualiquantitativa, descritivo, com aplicação de questionário autoaplicável, estruturado e eletrônico para o levantamento dos dados. O questionário foi aplicado entre fevereiro e março de 2021. O projeto foi aprovado pelo Comitê de Ética em Pesquisa do Hospital Israelita Albert Einstein (HIAE), com parecer: 4.528.519, CAAE: 40071220.0000.0071. Todos os participantes confirmaram o aceite em participar do estudo por meio de um Termo de Consentimento Livre e Esclarecido (TCLE) disponibilizado pela Internet, e o respondente recebeu uma via assinada, datada e rubricada pelo pesquisador responsável em seu endereço eletrônico.

Profissionais que trabalhavam com transplante de fígado no Brasil foram abordados virtualmente por meio do envio de *e-mail* e mensagens. O questionário da pesquisa foi disponibilizado pela Internet.

O critério de inclusão dos participantes no estudo foi o profissional de saúde trabalhar com o transplante de fígado em centros que utilizavam doadores de órgãos falecidos. Os critérios de exclusão foram profissionais que trabalhassem exclusivamente com doadores vivos e aqueles que não participavam do processo de escolha dos órgãos para transplante.

### Questionário estruturado

O questionário foi criado no programa *SurveyMonkey*^®^ 14 e estruturado com 17 questões distribuídas em quatro seções (Apêndice 1): Seção I – Fatores demográficos; Seção II – Percepção sobre a utilização de fígados de doadores falecidos; Seção III – Das razões para o descarte de fígados de doadores para transplante; e Seção IV – Das medidas para favorecer a utilização de fígados de doadores. As questões fechadas e de múltipla escolha abordaram as razões consensuais estabelecidas para a análise dos desfechos pesquisados e compuseram a porção quantitativa do estudo. Perguntas com campos abertos, nos quais o participante pode relatar outros dados, foram incluídas no questionário, para sua porção qualitativa.

Optou-se pela coleta de dados por meio de formulário eletrônico aplicado por meio da Internet objetivando favorecer a abrangência nacional da pesquisa, e para facilitar o contato com os participantes, bem como oferecer a eles a possibilidade de responder ao questionário em momento oportuno.

### Análise estatística

As variáveis categóricas foram descritas e analisadas utilizando-se número absoluto e frequência (percentagem). Para os dados qualitativos, os resultados foram apresentados na forma de relatórios que enfocaram o ponto de vista dos entrevistados. Os testes de análise estatística foram realizados utilizando-se o software (SPSS), versão 22 (IBM Corp, Armonk, New York).

## RESULTADOS

### Dados demográficos dos respondentes

Responderam à pesquisa 42 participantes. A maioria dos participantes (26; 61,90%) trabalhava em centros transplantadores na Região Sudeste do país e tinha mais de 5 anos de atuação no campo de transplante hepático (38; 90,48%). Ainda, os centros transplantadores aos quais os respondentes pertenciam majoritariamente realizavam mais de 60 transplantes por ano (24; 57,14%). A distribuição demográfica detalhada dos respondentes é apresentada na tabela 1.

**Tabela 1:** Características demográficas dos 42 participantes do estudo

Características		n (%)
Região do centro transplantador		
	Norte	2 (4,76)
	Nordeste	7 (16,67)
	Sul	6 (14,29)
	Sudeste	26 (61,90)
	Centro-Oeste	1 (2,38)
Trabalho com transplante de fígado, anos		
	<5	4 (9,52)
	5-10	13 (30,95)
	10 -15	10 (23,81)
	>15	15 (35,72)
Transplantes/ ano no serviço		
	<30	11 (26,19)
	30-60	7 (16,67)
	>60	24 (57,14)

### Percepção da utilização de fígados de doadores falecidos

Quando questionados acerca de sua percepção da taxa de utilização de fígados de doadores falecidos para transplante no Brasil (relação entre o número de órgãos oferecidos e o de transplantados), a absoluta maioria dos participantes (38; 90%) acreditava que esta era baixa ou muito baixa (Figura 1). Todos os participantes responderam ser possível aumentar a taxa de utilização de fígados de doadores falecidos no país.

**Figura 1:** Opinião dos respondentes quanto à sua percepção da taxa de utilização de fígado de doadores falecidos (relação entre o número de órgãos oferecidos e o número de órgãos transplantados) no Brasil

A maioria dos participantes (36; 85,71%) acreditava que, ao longo dos últimos anos, foi maior a prevalência de critérios de marginalidade entre os doadores de órgãos oferecidos para transplante, sendo que 9,52% (n=4) disseram que não e 4,76% (n=2) que estavam em dúvida. Em paralelo, 72% (n=30) dos participantes acreditavam que a expansão da população de doadores de critérios estendidos impactou negativamente a taxa de utilização dos órgãos. Os dados são apresentados na figura 2.

**Figura 2:** Percepção dos participantes quanto à influência negativa da expansão da população de doadores de critérios estendidos na taxa de utilização de fígados de doadores falecidos

### Razões para o descarte de fígados de doadores falecidos para transplante

Os participantes foram também questionados sobre qual seria a principal categoria de razões para a recusa de um órgão para transplante. A maioria dos respondentes (36; 85,37%) considerou as condições relacionadas com o doador (dúvidas em relação à função, idade, obesidade, etc.) como a principal categoria. A figura 3 apresenta todas as categorias questionadas, bem como a distribuição das respostas dos participantes.

**Figura 3:** Respostas dos participantes quanto às principais categorias de razões para a recusa de um fígado de doador falecido para transplante

Na sequência, eles foram arguidos sobre a frequência na prática clínica dos motivos para o não aceite para transplante de um fígado de doador falecido. Dezenove participantes (46%) selecionaram achados durante a cirurgia de extração (esteatose, anormalidades anatômicas, lesões durante a extração, perfusão insatisfatória do órgão e tamanho do órgão – desproporção doador/receptor) como o primeiro motivo. Problemas relacionados com o tempo de isquemia fria e a dificuldade para encontrar uma boa correspondência entre doador e receptor foram, na sequência, os motivos mais frequentes (Tabela 2).

**Tabela 2:** Principais motivos na prática clínica selecionados pelos participantes, por ordem de frequência, para a recusa de um fígado de doador falecido

Motivo	n (%)
Achados durante a cirurgia de extração (esteatose, anormalidades anatômicas, lesões durante a extração, perfusão insatisfatória do órgão e tamanho do órgão – desproporção doador/receptor)	19 (46,0)
Tempo estimado de isquemia fria prolongado (pela longa distância do hospital da captação do órgão até o centro transplantador)	14 (34,0)
Dificuldade para realizar uma boa correspondência entre doador e receptor (balanço entre a severidade da doença hepática do receptor e as características do doador/órgão)	15 (37,0)

Outras razões não incluídas nas opções do questionário e que foram destacadas pelos participantes foram a dificuldade de interpretação das informações do órgão devido à inexperiência do profissional responsável pela cirurgia de extração, a instabilidade hemodinâmica do doador, a dificuldade de obtenção de logística aérea para o transporte do fígado do doador e as condições infectocontagiosas ativas no doador (incluindo sorologia positiva para doença de Chagas).

### Medidas para favorecer a utilização de fígados de doadores falecidos

Os participantes foram questionados sobre a relação, em ordem de relevância, de possíveis medidas para otimizar a utilização de fígados de doadores falecidos para transplante. Das medidas sugeridas aos participantes, a melhoria no treinamento das equipes das unidades de terapia intensiva (UTI) nos cuidados com os doadores de órgãos foi listada como a mais relevante por 53,66% (n=22) dos respondentes. O desenvolvimento/investimento em novas tecnologias foi a segunda medida. Os resultados são expressos na tabela 3.

**Tabela 3:** Relação, por ordem de relevância, segundo os participantes, das principais medidas para otimizar a utilização de fígados de doadores falecidos

Medida	n (%)
Primeira medida: melhor treinamento das equipes das unidades de terapia intensiva nos cuidados com os doadores de órgãos	22 (53,66)
Segunda medida: desenvolvimento/investimento em novas tecnologias para melhorar a preservação e realizar a avaliação da função de órgãos de doadores antes do transplante (máquina de perfusão hepática)	22 (53,66)

Em relação às possíveis novas tecnologias para favorecer uma maior utilização de fígados de doadores, os participantes foram questionados em relação à sua percepção sobre um possível impacto positivo da máquina de perfusão hepática. A maioria deles (31; 75,61%) acreditava nesse dispositivo, sendo que 12,20% (n=5) responderam negativamente e 12,20% (n=5) selecionaram que não tinham uma opinião formada.

Vinte e sete (65,85%) participantes selecionaram existir outras estratégias não listadas nas opções que poderiam contribuir para a otimização da taxa de utilização de fígados de doadores falecidos. As opções sugeridas foram: realização das cirurgias de extração por profissionais experientes (principalmente nos doadores de critérios estendidos); criação de uma lista alternativa dedicada a pacientes oncológicos para órgãos descartados por todas as equipes; maior disponibilidade de recursos para a realização de biópsia hepática no hospital do doador; diminuição do tempo de consulta para avaliação do órgão pelas equipes; estabelecimento de uma política nacional para utilização de órgãos bipartidos; estabelecimento de auditorias das cirurgias de extração e das causas de descarte de órgãos com definição de critérios para registro e manutenção de centros transplantadores; facilitação da logística aérea para o transporte do fígado do doador, e promoção de um melhor entendimento sobre o balanço de riscos entre doador e receptor, que devia favorecer a utilização dos órgãos de doadores de maior risco.

## DISCUSSÃO

O desenvolvimento e a implementação de estratégias para melhorar a taxa de utilização de órgãos de doadores falecidos são fundamentais para permitir que um maior número de pessoas tenha suas vidas salvas e transformadas por meio do transplante de fígado. Neste estudo do tipo questionário envolvendo profissionais que trabalhavam com o transplante hepático, encontrou-se que a maioria dos profissionais considerava a taxa de utilização de fígados de doadores falecidos no Brasil baixa ou muito baixa, e, de forma unânime, eles concordaram que era possível aumentá-la. Condições relacionadas ao doador foi a categoria mais frequente de razões para a recusa de um fígado para transplante, sendo os achados durante a cirurgia de extração o motivo mais frequente na prática clínica. O melhor treinamento das equipes das UTI nos cuidados com os doadores de órgãos e o investimento em novas tecnologias para otimizar a preservação/avaliar a qualidade dos órgãos antes do transplante foram as medidas mais consideradas para ampliar com segurança a taxa de utilização de órgãos de doadores falecidos.

Recente estudo analisou dados do *Scientific Registry of Transplant Recipients* (SRTR). Nele, Carpenter et al., identificaram que fígados não transplantados eram mais frequentemente de doadores com idades mais avançadas, com maiores IMC, portadores de hepatites virais (vírus B e C) e que apresentavam maior número de comorbidades, revelando dúvidas em relação à qualidade do órgão.^(5)^ Nessa análise de 6.454 fígados de doadores após morte encefálica descartados para transplantes, a principal razão para descarte foram os achados de biópsia, seguido de “outros”: dúvidas da função do órgão, alterações anatômicas dos órgãos, piora clínica da doença do receptor, tempo prolongado de isquemia fria e falta de um receptor compatível.^(5)^

Semelhantes achados foram reportados em outro estudo, utilizando a base de dados da *United Network for Organ Sharing* (UNOS), com 9.021 fígados descartados.^(9)^ A causa mais comum para descarte dos órgãos de doadores falecidos para transplante foram os achados na biópsia (4.069 órgãos). Essa razão foi seguida por “outras razões específicas”, reportada para 1.456 órgãos. Os autores destacaram que, considerando que essas leituras são comumente realizadas por patologistas não especializados, a padronização dos relatórios pode permitir um aumento na taxa de utilização desses órgãos. Outras razões para o não aceite de um órgão foram: impossibilidade de encontrar um receptor adequado, história social, sorologia positiva para citomegalovírus ou hepatites virais, história médica do doador, ausência de condição clínica do doador, lesões vasculares, traumas e anormalidades anatômicas.^(9)^

Recentemente, um estudo retrospectivo de centro único realizado no Brasil revisou dados de 67 fígados de doadores falecidos descartados para transplante entre 2015 e 2018 após cirurgia de extração.^(10)^ Problemas relacionados com o órgão do doador foi a causa para a não utilização de metade dos órgãos (alterações macroscópicas patológicas, dano visível ao órgão e tamanho inapropriado), sendo que 36% foram associados com características clínicas e laboratoriais do doador (sorologia positiva para o vírus hepatite B, infecção no doador, antecedentes mórbidos do doador, uso prolongado de vasopressores e instabilidade hemodinâmica). Seis órgãos (9%) foram descartados por razões logísticas e tempo de isquemia fria.^(10)^

Os relatórios do Registro Brasileiro de Transplantes (RBT) de 2018 e 2019 reportam as doenças cerebrovasculares como a principal causa de óbito (55%) na população de doadores falecidos – e não mais as causas traumáticas.^(1,11)^ Também, destaca-se que 47% desses doadores têm mais de 50 anos, e, em 2019, houve aumento de 62,5% na taxa de doadores acima de 65 anos.^(1,11)^ Como discutido, esses doadores que extrapolam os padrões de um doador ideal são conhecidos como doadores de critério estendido. A prevalência desses doadores de alto risco tem aumentado ao longo dos anos como consequência da mudança demográfica nessa população, e, cada vez mais, eles são ofertados para transplante.^(12)^ Os doadores estão mais velhos, apresentam frequentemente obesidade e doenças crônicas e falecem por doenças cerebrovasculares, evidência também encontrada em estudos internacionais.^(3,4)^ Esses órgãos apresentam maior risco de complicações pós-operatórias e, consequentemente, com mais frequência, são recusados para transplante.^(4,12)^

A complexidade do potencial doador de órgãos é amplamente reconhecida como um desafio no cuidado intensivo, considerando a multiplicidade de suas prioridades fisiológicas para que permaneçam elegíveis para doação, no qual a estabilidade hemodinâmica é o principal fator de sucesso.^(13)^ Em vista disso, uma força-tarefa de sociedades e associações ligadas ao transplante de órgãos no território brasileiro buscou elaborar e difundir protocolos para o manejo do doador potencial em UTI.^(14)^ Embora crucial, a otimização do cuidado do potencial doador não pode alterar características biológicas do paciente, como idade, peso e comorbidades, e nem reverter agressões prévias aos órgãos.

O presente estudo retrata a necessidade de se expandir a utilização de fígados de doadores falecidos para transplante no Brasil, sendo que esta é comprometida pela prevalência de doadores de critérios estendidos. Ainda, o principal motivo para sua recusa foram os achados durante a cirurgia de retirada no doador, que geram dúvidas em relação à qualidade do órgão. Diante desse cenário, os participantes consideraram o desenvolvimento/investimento em novas tecnologias para melhorar a preservação dos órgãos e realizar a avaliação de sua função antes do transplante (máquina de perfusão hepática) uma medida que poderia aumentar essa taxa de utilização. A preservação dinâmica de órgãos, por meio da máquina de perfusão hepática, oferece preservação superior para órgãos de doadores de critérios estendidos e possibilita o recondicionamento e a avaliação da função dos órgãos antes do transplante.^(7,15,16)^ O fluxo contínuo de uma solução oxigenada através da vasculatura previne o dano isquêmico ao órgão, remove metabólitos tóxicos e permite a avaliação de sua atividade metabólica.^(17)^ Atualmente, essa tecnologia encontra-se em implementação para uso clínico em países como Reino Unido, Suíça e Espanha.^(15,16,18)^ No Brasil, a seleção cuidadosa de doadores e o conhecimento técnico-científico adequado podem garantir a implementação efetiva e satisfatória dessa tecnologia.^(19)^

Entre as estratégias para aumentar a utilização de órgãos de doadores falecidos propostas pelos participantes destacam-se melhorias na captação dos órgãos dos doadores, como a alocação de profissionais experientes para esse procedimento (principalmente nos doadores de critério estendido) e a facilitação da realização de biópsia hepática no hospital do doador e de logística aérea para o transporte do órgão. Outras iniciativas sugeridas se concentram no campo das políticas de transplantação, criação de uma lista alternativa dedicada a pacientes oncológicos para órgãos descartados por todas as equipes, diminuição do tempo de consulta para avaliação do doador, criação de uma política nacional de utilização de órgãos bipartidos e realização de auditorias das cirurgias de extração e das causas de descarte de órgãos, com definição de critérios para registro e manutenção de centros transplantadores. Finalmente, a disseminação do conceito de balanço de riscos entre doador e receptor deve favorecer a utilização dos órgãos de doadores de maior risco.

Certamente todas as medidas sugeridas são de grande valia e devem favorecer a otimização da taxa de utilização de fígados de doadores falecidos. Embora muitas dessas iniciativas dependam de entidades regulatórias e de financiamento externo, simples atitudes das equipes de transplante, como a designação de profissionais experientes para a cirurgia de retirada e a rápida avaliação das ofertas de doadores, podem favorecer a utilização desses órgãos. A criação de grupos de suporte ao acesso de novas tecnologias e de educação em transplante são outros exemplos de medidas benéficas. Assegurar o suporte e os recursos de que as equipes de transplante necessitam é fundamental para aumentar a utilização de órgãos de doadores falecidos, principalmente daqueles de maior risco.

Dentre as limitações deste estudo está o número de participantes, embora sua distribuição tenha sido representativa da localização dos centros transplantadores pelas regiões geográficas brasileiras. Como forma de mitigar a limitação advinda da aplicação de um questionário com respostas fechadas de múltipla escolha, o estudo apresentou um desenho qualiquantitativo, permitindo que os participantes oferecessem respostas em campos abertos.

## CONCLUSÃO

Entre profissionais de saúde diretamente envolvidos com o transplante de fígado no Brasil, há a percepção de baixa taxa de utilização de órgãos de doadores falecidos. O comprometimento desse índice de aproveitamento pode ser consequência de condições relacionadas aos doadores. Aumentar com segurança a taxa de utilização de órgãos é fundamental para salvar mais vidas. O compromisso e o trabalho em conjunto das equipes envolvidas no processo de doação e transplante com esse propósito comum são fundamentais para atingir essa meta. A melhora do cuidado intensivo com os doadores de órgãos e a implementação de novas tecnologias que possibilitem preservação superior e avaliação da qualidade de fígados de doadores de critérios estendidos antes do transplante foram indicadas neste estudo como as medidas mais relevantes.

## Apêndice 1 Questionário

**Table:**

Seção I – Fatores demográficos	
1. Em qual região do Brasil o seu centro transplantador está localizado?	
	a) Norte
	b) Nordeste
	c) Sul
	d) Sudeste
	e) Centro-Oeste
2. Há quantos anos trabalha especificamente com transplante de fígado?	
	a) <5 anos
	b) 5-10 anos
	c) 10-15 anos
	d) >15 anos
3. Quantos transplantes de fígado são realizados anualmente em seu centro?	
	a) <30
	b) 30-60
	c) >60
Seção II – Percepção sobre a utilização de fígados de doadores falecidos	
4. Você considera a taxa de utilização de fígados de doadores cadavéricos (relação entre o número de órgãos oferecidos e o número de órgãos transplantados) hoje no Brasil:	
	a) Alta.
	b) Adequada (todos os órgãos possíveis são utilizados).
	c) Baixa (seria possível otimizar a utilização de órgãos de doadores).
	d) Muito baixa (muitos órgãos que poderiam ser utilizados são descartados).
5. Você considera possível aumentar a taxa de utilização de fígados de doadores cadavéricos atualmente praticada no Brasil?	
	a) Sim
	b) Não
6. Você acredita que ao longo dos últimos anos tem havido uma maior prevalência de critérios de marginalidade entre os doadores de órgãos oferecidos para transplante?	
	a) Sim
	b) Não
7. Você acredita que a expansão da população de doadores de critério estendido tem afetado diretamente a taxa de utilização de fígados de doadores?	
	a) Sim
	b) Não
Seção III – Das razões para o descarte de fígados de doadores para transplante	
8. Mais comumente as suas razões para você não aceitar um órgão de doador para transplante se enquadram dentro de qual categoria abaixo?	
	a) Condições relacionadas com o doador (por exemplo: dúvidas em relação a função, idade e obesidade).
	b) Condições relacionadas com o centro transplantador (por exemplo: logística e falta de hemocomponentes).
	c) Condições relacionadas com o receptor (por exemplo: severidade da doença hepática do receptor).
9. Entre os motivos abaixo para não aceitar um órgão (fígado) de um doador cadavérico, qual você considera o principal em sua prática clínica?	
	a) Tempo estimado de isquemia fria prolongado (pela longa distância do hospital da captação do órgão até o centro transplantador).
	b) Dificuldade para realizar uma boa correspondência entre doador e receptor (balanço entre a severidade da doença hepática dos receptores e as características do doador/órgão).
	c) Dados da história clínica do doador (incluindo exames laboratoriais) – dúvidas em relação à função do órgão.
	d) Achados durante a cirurgia de extração (esteatose, anormalidades anatômicas, lesões durante a extração, perfusão insatisfatória do órgão e tamanho do órgão – desproporção doador/receptor).
	e) Achados da biópsia do órgão do doador.
10. Entre os motivos abaixo para não aceitar um órgão (fígado) de um doador cadavérico, qual você considera a segunda principal em sua prática clínica?	
	a) Tempo estimado de isquemia fria prolongado (pela longa distância do hospital da captação do órgão até o centro transplantador).
	b) Dificuldade para realizar uma boa correspondência entre doador e receptor (balanço entre a severidade da doença hepática dos receptores e as características do doador/órgão).
	c) Dados da história clínica do doador (incluindo exames laboratoriais) – dúvidas em relação à função do órgão.
	d) Achados durante a cirurgia de extração (esteatose, anormalidades anatômicas, lesões durante a extração, perfusão insatisfatória do órgão e tamanho do órgão – desproporção doador/receptor).
	e) Achados da biópsia do órgão do doador.
11. Entre os motivos abaixo para não aceitar um órgão (fígado) de um doador cadavérico, qual você considera a terceira principal em sua prática clínica?	
	a) Tempo estimado de isquemia fria prolongado (pela longa distância do hospital da captação do órgão até o centro transplantador).
	b) Dificuldade para realizar uma boa correspondência entre doador e receptor (balanço entre a severidade da doença hepática dos receptores e as características do doador/órgão).
	c) Dados da história clínica do doador (incluindo exames laboratoriais) – dúvidas em relação à função do órgão.
	d) Achados durante a cirurgia de extração (esteatose, anormalidades anatômicas, lesões durante a extração, perfusão insatisfatória do órgão e tamanho do órgão – desproporção doador/receptor).
	e) Achados da biópsia do órgão do doador.
12. Em sua opinião, há alguma outra razão para não aceitar um órgão (fígado) de um doador falecido que não foi listada nas opções acima? Se sim, por favor, especifique.	
Resposta___________________________________________	
Seção IV – Das medidas para favorecer a utilização de fígados de doadores falecidos	
13. Entre as medidas abaixo, qual você acredita que seria a principal para otimizar a utilização de fígados de doadores cadavéricos? (Selecione duas alternativas)	
	a) Ofertas apenas de doadores dentro de critérios mais restritivos (por exemplo: limite máximo de idade, nível sérico de sódio dentro ou próximo dos limites de normalidade e limites de índice de massa corporal).
	b) Desenvolvimento de um sistema de alocação de órgãos que já considere características dos doadores e receptores antes da oferta.
	c) Desenvolvimento/investimento em novas tecnologias para melhorar a preservação e realizar a avaliação da função de órgãos de doadores antes do transplante (máquina de perfusão hepática).
	d) Melhor treinamento das equipes das unidades de terapia intensiva nos cuidados com os doadores de órgãos.
	e) Alocação de órgãos apenas regionalmente (zonas de alocação), impondo limites de distância para a oferta dos órgãos.
14. Entre as medidas abaixo, qual você acredita que seria a segunda principal para otimizar a utilização de fígados de doadores cadavéricos? (Selecione duas alternativas)	
	a) Ofertas apenas de doadores dentro de critérios mais restritivos (por exemplo, limite máximo de idade, nível sérico de sódio dentro ou próximo dos limites de normalidade e limites de índice de massa corporal).
	b) Desenvolvimento de um sistema de alocação de órgãos que já considere características dos doadores e receptores antes da oferta.
	c) Desenvolvimento/investimento em novas tecnologias para melhorar a preservação e realizar a avaliação da função de órgãos de doadores antes do transplante (máquina de perfusão hepática).
	d) Melhor treinamento das equipes das unidades de terapia intensiva nos cuidados com os doadores de órgãos.
	e) Alocação de órgãos apenas regionalmente (zonas de alocação), impondo limites de distância para a oferta dos órgãos.
15. Você acredita que a implementação da máquina de perfusão hepática *ex situ* irá impactar positivamente o transplante de fígado, favorecendo uma maior utilização de fígados de doadores?	
	a) Sim
	b) Não
	c) Não tenho uma opinião formada.
16. Há outras estratégias que você considera que poderiam melhorar ainda mais a taxa de utilização de doadores?	
	a) Sim
	b) Não
17. Se você respondeu sim à questão anterior, por favor especifique qual estratégia você acredita que também contribuiria:	
Resposta___________________________________________
